# A Retrospective Analysis of the First Clinical 5DCT Workflow

**DOI:** 10.3390/cancers17030531

**Published:** 2025-02-05

**Authors:** Michael Lauria, Minji Kim, Dylan O’Connell, Yi Lao, Claudia R. Miller, Louise Naumann, Peter Boyle, Ann Raldow, Alan Lee, Ricky R. Savjani, Drew Moghanaki, Daniel A. Low

**Affiliations:** 1 Department of Radiation Oncology, University of California, Los Angeles, Los Angeles, CA 90095, USA; mkim3@mednet.ucla.edu (M.K.); doconnell@mednet.ucla.edu (D.O.); claudiamiller@mednet.ucla.edu (C.R.M.); lnaumann@mednet.ucla.edu (L.N.); pboyle@mednet.ucla.edu (P.B.); araldow@mednet.ucla.edu (A.R.); alalee@mednet.ucla.edu (A.L.); rrsavjani@mednet.ucla.edu (R.R.S.); dmoghanaki@mednet.ucla.edu (D.M.); dlow@mednet.ucla.edu (D.A.L.); 2 Department of Radiation Oncology, City of Hope, Duarte, CA 91010, USA; yilao@coh.org

**Keywords:** lung radiotherapy, 4DCT, model-based CT, motion management, image-guided radiotherapy

## Abstract

Background/Objectives: 5DCT was first proposed in 2005 as a motion-compensated CT simulation approach for radiotherapy treatment planning to avoid sorting artifacts that arise in 4DCT when patients breathe irregularly. Since March 2019, 5DCT has been clinically implemented for routine use at our institution to leverage this technological advantage. The clinical workflow includes a quality assurance report that describes the output of primary workflow steps. This study reports on the challenges and quality of the clinical 5DCT workflow using these quality assurance reports. Methods: We evaluated all thoracic 5DCT simulation datasets consecutively acquired at our institution between March 2019 and December 2022 for thoracic radiotherapy treatment planning. The 5DCT datasets utilized motion models constructed from 25 fast-helical free-breathing computed tomography (FHFBCTs) with simultaneous respiratory bellows signal monitoring to reconstruct individual, user-specified breathing-phase images (termed 5DCT phase images) for internal target volume contouring. Each 5DCT dataset was accompanied by a structured quality assurance report composed of qualitative and quantitative measures of the breathing pattern, image quality, DIR quality, model fitting accuracy, and a validation process by which the original FHFBCT scans were regenerated with the 5DCT model. Measures of breathing irregularity, image quality, and DIR quality were retrospectively categorized on a grading scale from 1 (regular breathing and accurate registration/modeling) to 4 (irregular breathing and inaccurate registration/modeling). The validation process was graded according to the same scale, and this grade was termed the suitability-for-treatment-planning (STP) grade. We correlated the graded variables to the STP grade. In addition to the quality assurance reports, we reviewed the contour sessions to determine how often 5DCT phase images were used for treatment planning and delivery. Results: There were 169 5DCT simulation datasets available from 156 patients for analysis. The STP was moderately correlated with breathing irregularity, image quality, and DIR quality (Spearman coefficients: 0.26, 0.30, and 0.50, respectively). Multiple linear regression analysis demonstrated that STP was correlated with regular breathing patterns (*p* = 0.008), image quality (*p* < 0.001), and better DIR quality (*p* < 0.001). 5DCT datasets were used for treatment planning in 82% of cases, while in 12% of cases, a backup image process was used. In total, 6% of image datasets were not used for treatment planning due to factors unrelated to the 5DCT workflow quality. Conclusions: The strongest association with STP was with DIR quality grades, as indicated by both Spearman and multiple linear regression analysis, implying that improvements to DIR accuracy and evaluation may be the best route for further improvement to 5DCT. The high rate of 5DCT phase image use for treatment planning showed that the workflow was reliable, and this has encouraged us to continue to develop and improve the workflow steps.

## Simple Summary

5DCT has been developed as a replacement for 4DCT that avoids sorting artifacts and provides quantitative motion characterization by employing a motion model for reconstruction. Since its development and clinical introduction in 2019, it has been the primary imaging modality for respiratory motion management in lung radiotherapy at our clinic, being used for more than 150 patients. The aim of this study is to report on our experience, including the frequency and magnitude of imaging artifacts, breathing irregularity, and image registration accuracy, correlating these quantities with 5DCT image reconstruction accuracy. We also report on the frequency of use of 5DCT images by physicians in the treatment planning workflow. This study is the first to report on the clinical implementation of 5DCT.

## 1. Introduction

Respiratory motion poses a significant challenge in radiation therapy, as its irregularity introduces uncertainty in tumor and normal organ positions that can compromise the precision of target contouring and the accuracy of treatment delivery [1,2]. The report of Task Group 76 (TG 76) of the American Association of Physicists in Medicine [3] addressed this challenge by describing clinical approaches for managing respiratory motion, including respiratory gating [4], breath-hold techniques [5], forced shallow-breathing methods [6], and respiration synchronization [7]. The TG 76 report also outlined simulation imaging solutions for motion management, including slow-scanning CT [8], breath-hold CT, and 4DCT [9]. Since the time of its publication (2006), the latter has offered the most promising solution for acquiring high-quality images for tumors with respiratory motion, despite its known limitations [10].

4DCT reconstructs three-dimensional images of lung tumors at several phases, defined by amplitude or time bins [11,12], of the breathing cycle by acquiring free-breathing scans, either with helical [9] or ciné [13] scanning. The image data are sorted into phase bins, and the sorted image data in each phase bin are then used to reconstruct 3D images. This process is conventionally completed for 8–10 phase bins so that the tumor can be tracked throughout the respiratory cycle. The phase-binned images are used to assess breathing motion and guide ITV contouring for treatment planning-based motion management [14].

Following recommendations of the AAPM [3,15], radiotherapy clinics have been using 4DCT to improve the management of respiratory motion for thoracic radiotherapy cases. A recent survey [16] of radiation therapy clinics published by AAPM’s TG 324 demonstrated that 93% of surveyed clinics employed 4DCT—a large growth since a previous survey [17] in 2009, which showed that at the time, only 40% of clinics had 4DCT capabilities. Despite its widespread availability and clinical use, in cases where patients exhibit irregular breathing, sorting artifacts can interfere with 4DCT image quality [18], obscuring tumor location(s), which could compromise motion assessment and lead to target coverage errors [19]. One study showed that 45 of 50 retrospectively analyzed patients simulated with 4DCT images had at least one sorting artifact, and that 6 of 20 patients with lung cancer had at least one sorting artifact visible within the tumor [20]. Though 4DCT has improved incrementally in recent years with its increasing adoption, fundamental issues remain with this technology that call for an alternative solution to further improve patient outcomes [10].

As an alternative to 4DCT, 5DCT has been developed to overcome sorting artifacts particularly in patients who breathe irregularly. 5DCT is a model-based CT (MBCT) technique introduced by Low et al. in 2005 [21]. MBCT approaches like 5DCT are driven by motion models, rather than relying on sorting projections or ciné images [22,23]. The 5DCT name stems from the motion model’s description of tissue displacement as a function of the following five variables: (1–3) x, y, and z position in the reference geometry; (4) breathing amplitude; and (5) breathing rate. The 5DCT model relates tissue displacement, measured by deformable image registration (DIR), to the breathing pattern, measured by a respiratory surrogate such as spirometry [24], surface imaging [25], or an abdominal bellows [26]. Once the motion is characterized by the 5DCT model, user-defined phase images can be reconstructed by applying the model to a designated reference scan. In our clinic, we first take the representative breathing waveform (as described in Section 2.2) from the 5th percentile to 95th percentile amplitudes and remove the outliers outside of this range. From the remaining waveform, we generate eight 5DCT phase images at 0th-, 25th-, 50th-, 75th-, and 100th-percentile amplitude during exhalation and 25th-, 50th-, and 75th-percentile amplitude during inhalation.

Early 5DCT characterizations were reported in 2013 and 2014 [2,27]. The initial publications described how 5DCT could be implemented with conventional CT equipment and protocols, DIR, and a universal breathing motion model equation to describe breathing motion on a voxel-by-voxel basis. A novel free-breathing image acquisition method leveraging fast-helical free-breathing CT (FHFBCT) acquisition was introduced in 2013 to optimize the quality of images needed for 5DCT. Eventually, 5DCT was compared to conventional 4DCT protocols and demonstrated to be more robust for irregular breathing, while producing artifact-free images with similar imaging doses [27].

**Figure 1:**
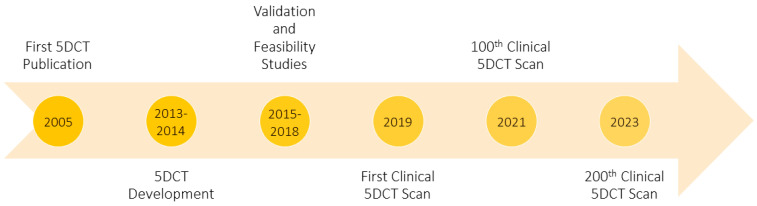
5DCT development and implementation timeline.

To validate the 5DCT workflow, O’Connell et al. first demonstrated that the 5DCT technique produces similar results as commercial 4DCT for simulating patients with regular breathing cycles [28]. They used mechanically ventilated pigs to generate images with 4DCT and 5DCT, evaluated landmarks throughout the lungs in the phase images, and identified position agreement within 2 mm for most cases. They later evaluated their workflows by comparing tumor motion, ITV generation, noise characteristics, and artifacts between both approaches, once again demonstrating consistent results using 5DCT with reduced artifacts [29]. Subsequent studies reported on the techniques needed to improve the quality and efficiency of 5DCT, such as de-blurring [30]. A historical summary of the 5DCT development and implementation timeline is shown in Figure 1.

Following early efforts to validate and prepare 5DCT for clinical use, the first clinical 5DCT scan was acquired for routine clinical care at our institution in March 2019, fourteen years after the initial publication [2]. Since 2019, more than 310 patients have been scanned clinically with 5DCT in place of 4DCT. In this study, we report on our initial clinical experience (up to December 2022) with 5DCT and evaluate patient and technical factors that can influence its quality.

## 2. Materials and Methods

The goal of this study was to report on our experience with the primary drivers limiting the fidelity of the clinical 5DCT phase image generation process. We performed a retrospective analysis of the consecutive patients simulated with a 5DCT protocol at our institution from March 2019 to December 2022, utilizing the patient-specific quality assurance (QA) reports to identify patient and technical factors associated with the suitability of the image sets for treatment planning.

### 2.1. 5DCT Protocol

**Figure 2:**
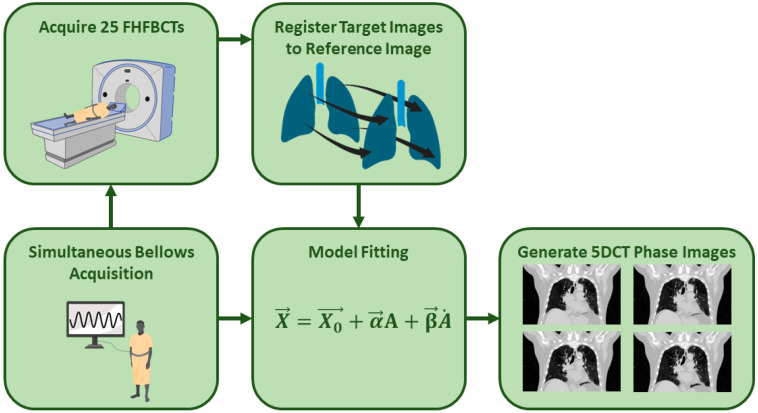
5DCT acquisition and reconstruction flowchart. Note that while the registration figure shows lungs, the registration and subsequent DVFs are acquired throughout the entire CT scan.

The 5DCT protocol in use at our institution relies on a motion model-based technique relating tumor displacement to breathing amplitude and rate as captured during the acquisition of 25 FHFBCTs. A schematic of the 5DCT workflow is shown in Figure 2, and further details can be found in prior publications [2,27]. To begin, 25 FHFBCTs are acquired with simultaneous respiratory monitoring using a sealed abdominal bellows (Lafayette Instrument Company, Lafayette, IN, USA) and a pressured transducer monitoring the internal bellows’ air pressure. Each slice of the FHFBCTs is assigned an uncorrected breathing amplitude and rate based on the bellows measurement and a time synchronization (offset) between the bellows and CT scanner. Due to the patient’s body temperature heating the air in the bellows, there is an inherent signal drift. This signal drift is corrected by optimizing a linear function that maximizes the correlation of the patient’s anterior abdominal skin surface position to the breathing amplitude.

The first FHFBCT is acquired using 140 mAs to serve as the reference scan. The other 24 FHFBCT scans, acquired with 40 mAs, are registered to the reference scan using DIR. The voxel-wise deformation vector fields (DVFs) thereby measure the breathing motion across the 25 images.

At each reference image voxel, including those outside the lungs, the 25 DIR voxel positions are fitted to their corresponding amplitudes and rates to fit the voxel- and patient-specific 5DCT motion model parameters. Equation (1) describes the model, where $\vec{X}$ is the voxel position at a breathing amplitude A and breathing rate $\dot{A}$; $\vec{X_{0}}$ is the voxel’s position in the reference image; $\vec{\alpha }$ is a voxel-specific model parameter that scales the breathing amplitude, A; and $\vec{\beta }$ is a voxel-specific model parameter that scales the breathing rate, $\dot{A}$. Note that, for tissue voxels that do not move, such as in the spine, both $\vec{\alpha }$ and $\vec{\beta }$ are zero. After fitting the 5DCT model parameters, Equation (1) is used to generate a vector field to deform the reference image and generate images at any user-defined breathing amplitude and rate (A and $\dot{A}$):

(1) $$\vec{X}=\vec{X_{0}}+\vec{\alpha }*A+\vec{\beta }*\dot{A}$$

### 2.2. Clinical Workflow

After the scans and bellows signals were acquired, the DIR and subsequent model generation were calculated on a computer server. The DIRs were performed using the deeds algorithm [31,32], while model reconstruction was performed in MATLAB (The Mathworks Inc., Natick, MA, USA). This process generally required several hours to complete, largely due to DIR processing, and provided the following image datasets used for treatment planning: (1) 8 5DCT phase images intended to replace the images provided by 4DCT in our 4DCT clinical workflow; (2) a maximum intensity projection (MIP) image, generated in MIM Vista software version 7.3.5 (MIM Software, Inc., Cleveland, OH, USA), of the 5DCT phase images; and (3) a maximum-intensity projection of the 25 FHFBCTs, also generated in MIM, termed the MEGA-MIP. The MEGA-MIP served as both a second check for the 5DCT tumor motion trajectory and a backup method when the 5DCT failed the quality checks described below.

**Figure 3:**
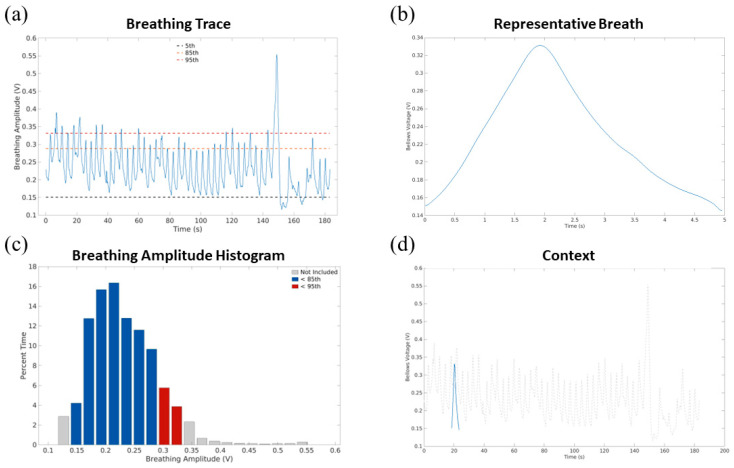
Example respiratory trace and subsequent representative breath for one patient, including (**a**) the respiratory trace annotated with the 5th, 85th, and 95th percentile amplitudes; (**b**) the representative breath resulting from this breathing trace; (**c**) the breathing amplitude histogram showing the percent of time spent in each amplitude bin; and (**d**) the representative breath in the context of the entire breathing trace.

The 5DCT phase images were based on breathing amplitudes and rates from a representative breath that was generated using the patient’s breathing trace, as described by White et al. [33]. Figure 3 shows examples of the annotated breathing waveform and representative breath for one patient.

In addition to the generation of the 5DCT phase images and MIPs, a process was also implemented to provide measures of 5DCT modeling and workflow validation that were easily interpretable. The first measure was the motion model fit residuals, reported as the distances between the model’s predicted voxel positions and the voxel positions acquired from the DIRs [28]. These were presented as summary statistics (mean, standard deviation, 95th percentile), histograms, and MIPs in the report. The second measure employed the 5DCT model and the breathing traces for the FHFBCTs to re-generate the original FHFBCTs using the reference image. This measure was termed the original scan reconstruction, which offered a comparison of 5DCT-generated images to the FHFBCT scans, considered by us as ground truth images.

The physicist assigned to the case was required to determine whether the 5DCT phase images could be used by the clinic for ITV contouring, generally relying on printed reports, described in the next section, to identify potential signs of catastrophic failures such as highly misregistered diaphragms or large (>~2 mm) residual errors. The assigned physicist also compared the 5DCT MIP to the MEGA-MIP and original 25 scans to determine whether the 5DCT reconstruction adequately reflected the tumor motion envelope. If the 5DCT failed to pass these quality checks, backup protocols were utilized, including the MEGA-MIP and reviewing the FHFBCTs as a movie sequence for treatment-planning purposes. While not providing a specific representative motion summary of the tumor, these images reflected the motion as characterized over the approximately 2 min CT scan acquisition period, although images taken during outlier breaths would not have been removed as their influence on the representative breath and, consequently, the 5DCT phase images would.

### 2.3. Quality Assurance (QA) Reports

Table 1 summarizes the contents of each QA report section, highlighting those that provided a qualitative summary of the breathing irregularity, FHFBCT quality, DIR accuracy, and modeling accuracy. The first section summarized FHFBCT acquisition, including a graph of the detected scan start and stop times and a display of the scan ranges. This section also included a display of the offset corrections and a summary of the drift correction process.

The second section of the QA report summarized the breathing pattern with plots annotating the 5th, 85th, and 95th percentiles; the segmentation of the breathing trace that was used to define the representative breath was also plotted. A breathing amplitude percentile histogram was also provided, along with plots of the representative breath alone and within the context of the entire breathing trace.

The third section of the QA report summarized the DIR results, relying on superimposed images of the reference FHFBCT and the registered target FHFBCTs in the mid-slice coronal, sagittal in the left lung, and sagittal in the right lung. Each overlay was presented as an image overlay, where the reference and target FHFBCTs were green and magenta, respectively. In locations where the images were aligned, the green and magenta colors created the typical gray scale. In the lungs, this was especially evident for the airways, blood vessels, and lung boundaries. In misaligned regions, the structures of one image would appear superimposed on the parenchyma of the other image and appear as either green or magenta.

The fourth section of the QA report summarized the motion modeling quality, evaluating model fit residuals that were summarized as mean, standard deviation, and 95th percentile and displayed as an MIP within the lungs, which had been masked using the Pulmonary Toolkit [34]. Image overlays were presented as in the DIR section, with the FHFBCT image in green and the 5DCT-generated image in magenta.

### 2.4. Review and Grading of QA Reports

**Figure 4:**
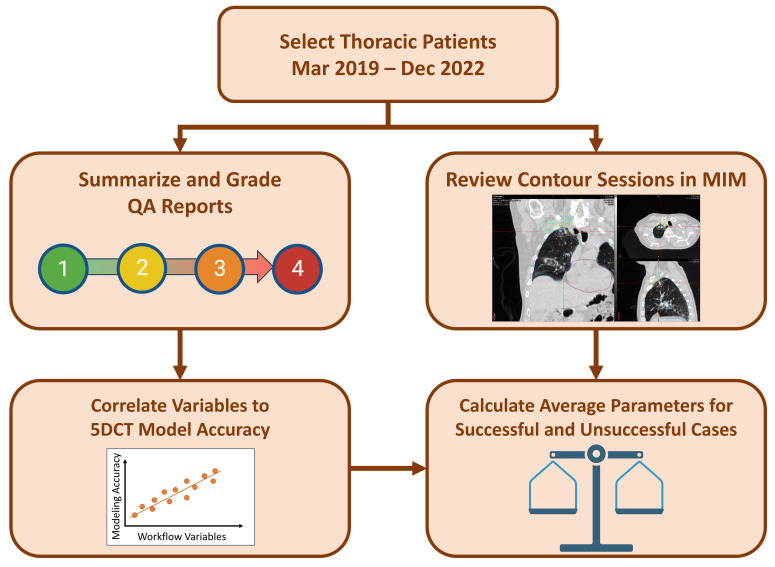
Flowchart of study design. MIM (MIM Software, Inc., Cleveland, OH, USA) was our RT-PACS system used for contouring.

**Figure 5:**
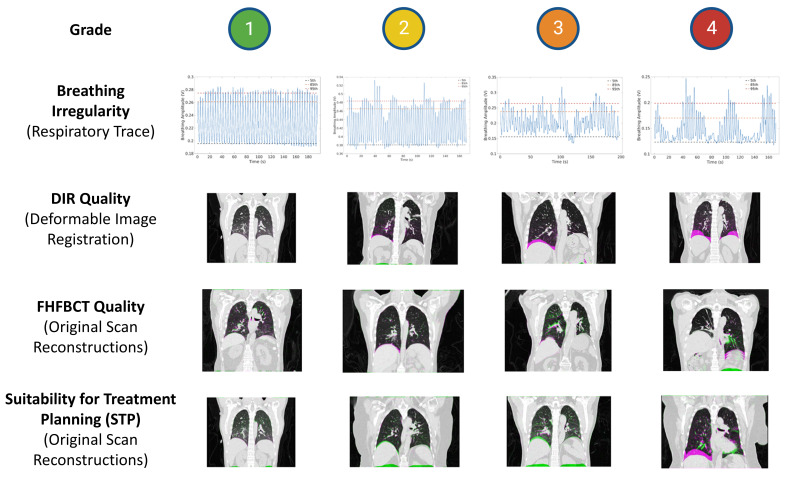
Examples of graded variables.

Figure 4 shows a flowchart summarizing the overall study design. We retrospectively employed a grading system to quantify several variables in the QA report, as well as to assess the suitability of the 5DCTs themselves for treatment planning. This grading system was adapted from a previous 5DCT validation study by O’Connell et al. [29]. Three workflow variables were graded—breathing irregularity, FHFBCT quality, and DIR quality—with the grades described in Table 2. Our FHFBCT and DIR quality reviews focused heavily on the diaphragm because we relied on the original FHFBCT scans prior to contouring, so the tumor location was not available to aid in quality analysis. The Suitability for Treatment Planning (STP) was graded on the same scale for the severity of discrepancies in the original scan reconstructions. Table 1 indicates which report components were used for each graded quantity. Figure 5 shows examples of the grades for each workflow variable.

### 2.5. Determination of 5DCT Clinical Use

We evaluated the outcomes of all 5DCT scans acquired in our department by their usability, which had been determined by the treatment-planning team, to assign them to one of seven categories, as summarized in Table 3. These categories were collected into three groups. Categories 1 and 2 were assigned whenever the 5DCT was clinically utilized. Categories 3 and 4 were assigned when the 5DCT phases were of insufficient quality for clinical use. Categories 5, 6, and 7 were assigned when no ITV was contoured, for example, when the patient was treated using our MR-linac (Category 5).

### 2.6. Statistical Analysis

We evaluated possible 5DCT modeling correlates and usability through three key indicators: (1) the STP grade, (2) model residual statistics (mean and 95th percentile), and (3) use in the clinic as defined in Table 3. We calculated Spearman correlation coefficients between each graded workflow variable and accuracy metric, with breathing irregularity grade, FHFBCT quality grade, and DIR quality grade as independent variables and STP grade, mean model residual, and 95th percentile model residual as dependent variables. Additionally, we calculated linear regression models to adjust for potential confounders. We performed multiple regression tests to assess STP grade as the dependent variable, with breathing irregularity grades, FHFBCT quality grades, and DIR quality grades as the independent variables. We calculated *p*-values for each regression, considering *p* < 0.05 to be statistically significant. We also calculated adjusted R^2^ values to assess the correlation strength. To assess the relationship between our criteria and the clinical utility, we calculated the mean STP grade and model residuals of 5DCT phase images that were used for treatment planning versus those that were not used, as categorized in Table 3.

## 3. Results

We identified 212 patients simulated with a 5DCT acquisition from March 2019 to December 2022. Of those, 56 patients had been scanned for abdominal tumors. For the purposes of this report, we focused only on the 156 patients imaged for thoracic tumors, including 169 unique 5DCT phase image sets (13 patients had two or more 5DCT scans). The mean age of the included patients was 67.7 ± 15.2 years. Of the patients, 45% were male and 55% were female.

**Figure 6:**
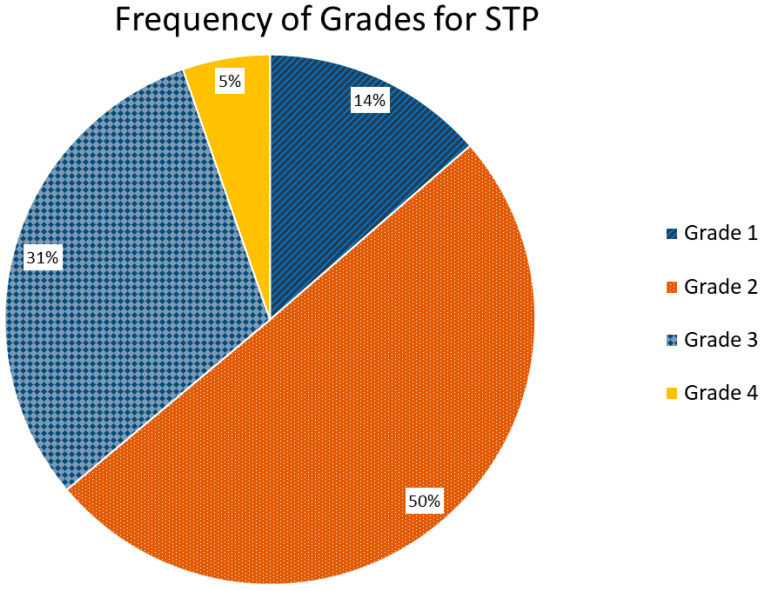
Pie-chart distribution of STP grade for all 5DCT image acquisitions (n = 169). Grades were assigned to indicate the alignment of the FHFBCTs and the model-generated FHFBCT according to whether they were “very good” (grade 1), “good” (grade 2), “poor” (grade 3), or “very poor” (grade 4). (STP: suitability for treatment planning; FHFBCT: fast-helical free-breathing computed tomography).

**Figure 7:**
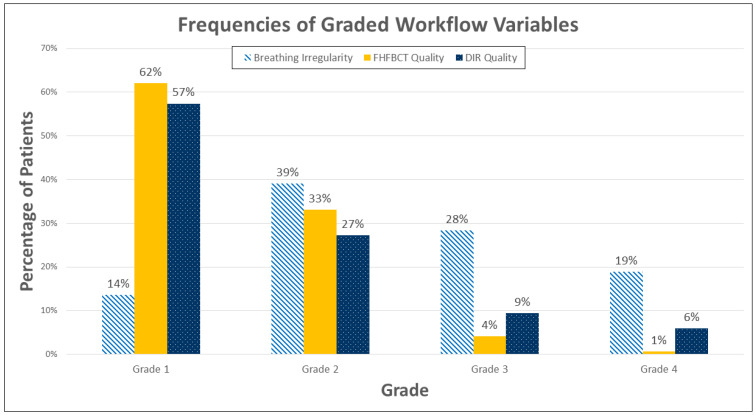
Histogram of grades for FHFBCT quality, DIR quality, and breathing irregularity across all cases.

STP grades were 1–4 (best to worse) in 14%, 50%, 31%, and 5% of cases, respectively (Figure 6). Breathing irregularity was graded 1–4 (best to worse) in 14%, 39%, 28%, and 19% of cases, respectively, indicating that patients commonly experienced an appreciable degree of breathing irregularity. FHFBCT image quality was graded 1–4 (best to worse) in 62%, 33%, 4%, and 1% of cases, respectively, indicating that artifacts were only “some” or “severe” in 5% of cases. DIR quality was graded 1–4 (best to worse) in 57%, 27%, 9%, and 6% of cases, respectively, indicating that poor or very poor image registration was uncommon. Figure 7 shows histograms of all investigated workflow variables.

Using Spearman correlations, we found that breathing irregularity grades (*p* = 0.008), FHFBCT quality grades (*p* < 0.001), and DIR quality grades (*p* < 0.001) were significant predictors of the STP grade. DIR quality showed the highest Spearman correlation at 0.50. Table 4 summarizes the Spearman correlation coefficients of the graded workflow variables and the STP grades for the analyzed scans. We found that the same predictors of STP grade were significant in the multiple linear regression model, with DIR grade again having the highest correlation. Table 5 shows these results (adjusted R^2^ = 0.378). Breathing irregularity had the highest correlation to the multiple linear regression model predicting mean residuals, while DIR quality had the highest correlation with the 95th percentile residual, suggesting that DIR quality may be more predictive of severe issues with model fitting. Table 6 shows the results for the multiple linear regression model predicting the mean model residual (adjusted R^2^ = 0.287). Table 7 shows the results for the multiple linear regression model predicting the 95th-percentile model residual (adjusted R^2^ = 0.319).

**Figure 8:**
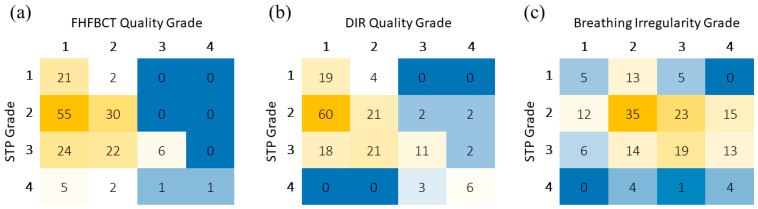
Heatmaps showing the coincidental frequency of (**a**) FHFBCT quality grade, (**b**) DIR quality grade, and (**c**) breathing irregularity grade with STP grades for all patients. The colors reflect the numbers displayed and are intended for assisted visual interpretation.

These relationships are visualized in Figure 8, showing heatmaps summarizing the coincidence of each grade between the STP and the three graded workflow variables. From these figures, the stronger relationship between DIR quality and STP can be seen compared to that of breathing irregularity. Figure 8a also shows that FHFBCT quality does not have a very strong relationship for Grades 1 and 2 but has a strong relationship for Grades 3 and 4.

**Figure 9:**
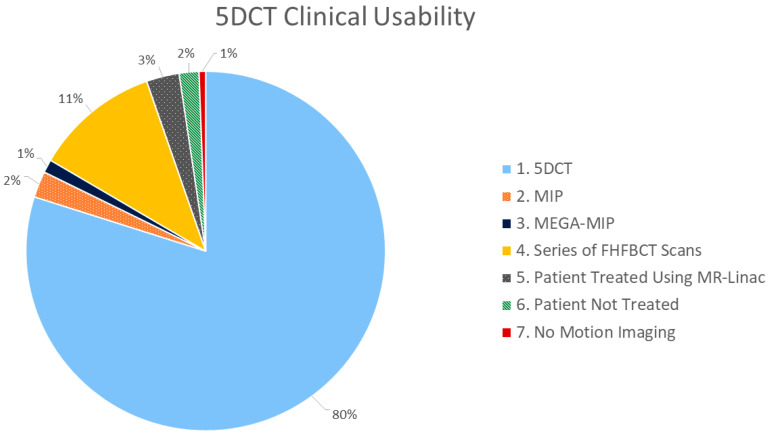
Pie chart summarizing the proportion of the 5DCT clinical usability categories identified.

Regarding clinical usability of the 5DCT images, 139 out of 169 patient scans were used by clinicians for contouring and treatment planning, while 21 utilized a backup protocol or the MEGA-MIP. Five patients were ultimately treated on the MR-linac due to excessive respiratory motion. Among the 139 patient scans that used 5DCT for contouring, 45 patient scans had high STP grades, indicating poor 5DCT reconstruction (38 with grades of 3; 7 with grades of 4). The average breathing irregularity grades for scans where 5DCT phase images were used for contouring versus the backup protocol were 2. 45 and 3.10, respectively, and their FHFBCT quality grades for each were 1.40 and 1.62, respectively. The DIR quality grades were 1.56 and 1.90 for each, respectively. The average STP grades for each were 2.22 and 2.62, respectively. The average mean residuals for each were 1.21 and 1.80, respectively. Figure 9 shows the breakdown of clinical uses of 5DCT reconstructions for contouring.

## 4. Discussion

The goal of this study was to report on clinical 5DCT and the factors that impact its use. 5DCT image sets acquired over a 33-month period were used for treatment planning for 82% of patients, with 12% requiring a backup protocol that utilized the same FHFBCT images acquired; no patient needed to return for a repeat CT simulation scan. These results demonstrate that 5DCT served as a replacement to 4DCT for thoracic radiotherapy treatment planning.

In total, 36% of scans had image reconstruction scores of 3 or 4, and we found that the biggest cause for these was poor DIR quality, as evidenced by Spearman correlation and multiple linear regression models. Image registration in the 5DCT workflow was, thus, a limiting aspect for modeling accuracy. This was an expected result as 5DCT was designed to be robust to irregular breathing, hence its major advantage over 4DCT, but largely relies on DIR to track tissue across the 25 FHFBCTs. 5DCT creates less reliability on breathing regularity and places more importance on DIR. This is important as DIR can be improved through advanced techniques and continued development, while breathing irregularity cannot for free-breathing protocols.

The findings of this study are the first reported evidence of 5DCT’s utility in the clinic, following the development and validation studies performed by Low et al. and O’Connell et al. from 2005 to 2018. Based on our findings, we are currently making improvements to the 5DCT workflow, along with using a new and faster CT scanner, and we will evaluate those improvements in follow-up studies. Now that we have evaluated the clinical efficacy of 5DCT, we can begin to study its clinical impact in terms of treatment planning quality and clinical endpoints.

As DIR quality was the primary driver of 5DCT STP, more accurate and robust image registration algorithms, especially ones that handle large motion magnitudes, could lead to the direct improvement of 5DCT. Some promising registration techniques on the horizon include deep-learning-based methods [35] or commercial and open-source options [36]. As an example, we have found that an updated version of deeds, deedsBCV, is more capable of registering patients with larger motion magnitudes [37], and we have implemented deedsBCV in our next iteration of the 5DCT workflow. Reviewing clinical 5DCT cases calls for further expertise to diagnose registration issues, so there is also a need for further developments in registration evaluation tools [38]. Our group has developed one such tool to quantify sub-millimeter DIR errors [39] that we will implement into the clinical QA process. In addition to improving DIR, we aim to improve the model through alternative surrogates that avoid the amplitude measurement signal drift. Wilms et al. showed that 1-D surrogates were mostly consistent in respiratory modeling accuracy, but that increasing the dimensionality of the surrogate led to great improvements [40]. We will thus test the feasibility of surface imaging as a 5DCT surrogate. Moreover, the deformed reference image serves to create anatomically accurate images, but the Hounsfield units are derived from the reference image and do not reflect the change in parenchymal tissue density during breathing. This does not greatly affect megavoltage photon radiation therapy but would introduce proton range errors for proton therapy. We are developing a ventilation adjustment process to scale the Hounsfield units to reflect local changes in air content using the principles of CT-based ventilation [41,42,43].

In addition to providing a reliable motion characterization for treatment planning, the 5DCT motion model could be used for downstream modeling applications. For example, there has been a large effort towards measuring ventilation with 4DCT-based motion models, but 4DCT sorting artifacts have been shown to impact these measurements [44]. 5DCT could be useful in enabling new methods for ventilation calculations with more highly sampled and robust data. Clinical trials have already shown the impact of using ventilation maps in treatment planning to reduce radiation pneumonitis [45,46], so increased accuracy with 5DCT could help further refine an already impactful technique. There is also a large research effort towards correcting motion artifacts in cone–beam CT with 4D-CBCT [47] or motion modeling [48,49], and we have demonstrated the potential of the 5DCT model in this area [50]. Its benefits are both immediate in delivering a sorting-artifact free CT simulation approach and extensive in biomechanical and daily imaging applications.

One limitation of our study was the restriction of retrospectively reviewing the QA report, requiring the manual grading process to analyze the workflow variables and STP. While we made efforts to standardize and guide the observer, for example, with the rubric shown in Figure 5, this process leaves room for observer bias. As we continue to improve the 5DCT process, future prospective studies will include more efforts to collect quantifiable data. Another limitation is the need to rely on the contouring sessions to determine whether and how 5DCT was used for a particular patient. Specifically, in the five MR-linac cases, we could not determine whether the 5DCT would have been used for contouring had the patient been treated on a conventional linac. It is likely that those scans were adequate to determine that there was too much motion for ITV planning, and that gating would greatly improve treatment efficacy.

## 5. Conclusions

5DCT simulation for radiation therapy treatment planning has been successfully implemented in our institution since 2019. Our analysis of the first 169 cases simulated with this technique demonstrates that 5DCT was clinically used in 82% of cases. We identified that, although 5DCT can generate robust images in the context of irregular breathing, there remains room for improvement in the DIR operational component to improve reconstructed image reliability. Our roadmap for continued development of the 5DCT workflow includes introducing modern DIR algorithms, implementing a quantitative DIR evaluation tool, scaling the HUs of model-derived scans to reflect ventilation, and exploring new 2D surrogates for the respiratory pattern. Our next evaluation study will address the direct impacts of 5DCT on the treatment planning workflow, including ITV generation and dosimetric differences between 4DCT and 5DCT. With these next steps, we anticipate increased modeling accuracy and a demonstration of the benefits of 5DCT for lung radiotherapy treatment planning.

## Figures and Tables

**Table 1:** Summary of data elements in 5DCT QA reports.

Data Element	Description	Purpose
Image and Breathing Surrogate Acquisition		
Scan Start/Stop Times	Binary signal of the CT on/off state, used to synchronize the scans to the bellows signal	This was used to assure that the noise level in the signal was not excessive, allowing for a clear determination of beam-on and beam-off times.
Scan Ranges	Plot of the slices available against the scan numbers, used to note if any slices or scans were missing from each scan	Some, especially early scan datasets, did not have common craniocaudal coverage due to CT sequence programming errors. These were rare, and if one of the 25 scans was too short, it was removed from further analysis by the physicist running the 5DCT protocol.
Corrected Shifts	Summary of any interpolations to correct offsets between head-first and foot-first scans	Even with careful programming, the scanner reconstructed the head-to-foot and foot-to-head with slightly different (sub-millimeter) couch positions. These were accounted for in the image processing, so this step was intended to assure that there were no >1 mm differences indicative of a CT programming error.
Bellows Signal/Abdominal Height	Summary figures of all detected abdomen heights and plot of the heights against the corresponding drift-corrected bellows signals, used to show the effectiveness of the drift correction and the correlation of the drift-corrected bellows signal and the abdomen heights.	This was used to assure that the correlation was high and did not have the appearance of randomness and to assure that the corresponding linear fit was a good representation of the data.
Respiratory Surrogate Analysis		
Respiratory Trace	Plot of the bellows amplitude signal over time with 5th-, 85th-, and 95th-percentile amplitudes annotated. Used to evaluate breathing irregularity in this study.	Examine the breathing trace to evaluate for obvious anomalies such as discontinuities or severe uncompensated drift.
Respiratory Amplitude Histogram	Histogram of time while the signal was in each amplitude bin	Not evaluated. This histogram was provided for retrospective review. There were and are no evaluation criteria tied to these types of data, and the respiratory trace contained a more easily evaluable form of these data.
Waveform Segmentation	Plot of the bellows amplitude signal over time with each detected exhalation point annotated, used to show how breaths were segmented to determine the representative breath.	Examined the exhalation points, which were used in the process of creating the representative breath.
Representative Breath and Context	Plots of the representative breath, alone and superimposed over the breathing waveform at its appropriate amplitudes.	This was used to assure that the representative breath amplitudes (peak inhalation and exhalation) reflected the overall breathing pattern.
Deformable Image Registration		
Deformable Image Registration	A set of 24 coronal, right lung sagittal, and left lung sagittal images, showing a green/magenta overlay of the reference image and deformed target FHFBCT at the middle slice of each plane. Used to evaluate DIR quality in this study.	Examined to determine if the image registration failed. This was generally due to the DIR algorithm’s inability to register images with large differences in breathing amplitudes.
Motion Modeling		
Summary	Summary table including number of scans, reference scan number, mean, standard deviation, and 95th percentile of the 5DCT model fit residuals.	Mean, standard deviation, and 95th percentile of the 5DCT model fit residuals were evaluated in conjunction with the 5DCT model fit residual histogram to determine if the 5DCT 8-phase images and 5DCT MIP should be used clinically.
Residual Histogram	Histogram of the 5DCT model residuals in 1 mm bins with frequency represented by percent of lung voxels	Used in conjunction with the mean, standard deviation, and 95th percentile of 5D model fit residuals to determine if the 5DCT 8-phase images and 5DCT MIP should be used clinically
5DCT Model Residual AP/Lat MIPs (mm)	Coronal and sagittal maximum-intensity projections (MIPs) of the model residuals overlain with a projection of the anatomy. Residuals were shown on a green-to-red color wash.	These were used to determine the magnitude and rough locations of the 5DCT model residual error distribution.
Original Scan Reconstructions	Coronal, left lung sagittal, and right lung sagittal overlays of the 5DCT model-deformed reference image superimposed with each of the 25 FHFBCTs using the green/magenta color overlay. Used to evaluate image quality and STP in this study.	Used in conjunction with the 5DCT motion model residuals to determine overall 5DCT workflow quality and clinical usability. Since no quantitative values were assigned to these images, they were primarily used to verify that high or low residual values accurately reflected poor or good original scan reconstructions, respectively.

**Table 2:** Grading criteria used to report the quality of breathing irregularity, fast-helical free-breathing computed tomography (FHFBCT) image quality, deformable image registration (DIR), and STP. Examples of artifacts are given in parentheses.

Variable	Grade 1	Grade 2	Grade 3	Grade 4
Breathing Irregularity	Very regular	Regular	Irregular	Very irregular
FHFBCT Image Quality	No artifacts	Minor artifacts (Some blurring at the diaphragm)	Some artifacts (Slight doubling of the diaphragm)	Severe artifacts (Severe doubling of the diaphragm)
DIR Quality	Great alignment of the reference and target FHFBCTs	Good alignment of the reference and target FHFBCTs (Minor misalignments that would not significantly impact modeling)	Poor alignment of the reference and target FHFBCTs (Some images are misaligned by greater than 1 mm at the diaphragm or vessels)	Very poor alignment of the reference and target FHFBCTs (Many or all images are misaligned by much more than 1 mm at the diaphragm or vessels)
Suitability for Treatment Planning	Great alignment of the true FHFBCTs and the model-generated FHFBCTs	Good alignment of the true FHFBCTs and the model-generated FHFBCTs (minor misalignments that signify minor errors in modeling)	Poor alignment of the true FHFBCTs and the model-generated FHFBCTs (some images are misaligned by greater than 1 mm at the diaphragm or vessels that signify some meaningful errors in modeling)	Very poor alignment of the true FHFBCTs and the model-generated FHFBCTs (many or all images are misaligned by much more than 1 mm at the diaphragm or vessels that signify meaningful errors in modeling)

**Table 3:** Categories of 5DCT clinical usability based on saved contouring sessions.

Category	Description
5DCT Used for ITV Contouring (N = 118)	
1	5DCT phase images reconstructions used for ITV contouring
2	MIP generated from 5DCT phase images reconstructions used for ITV contouring
Backup Protocols Used for ITV Contouring (N = 21)	
3	MEGA-MIP used for ITV contouring
4	Series of FHFBCT scans in sequence used for contouring
Other (No ITV Identified) (N = 30)	
5	Patient treated using an MR-linac (unrelated to 5DCT quality)
6	Patient not treated (unrelated to 5DCT quality)
7	No motion imaging used in contour session (due to lack of evidence of motion)

**Table 4:** Spearman correlation of graded workflow variables to the STP grades.

Correlation	Spearman Coefficient	Spearman Confidence Interval
Breathing irregularity Grade vs. Suitability for Treatment Planning Grade	0.260 (*p* < 0.001)	[0.109, 0.399]
Imaging FHFBCT Quality Grade vs. Suitability for Treatment Planning Grade	0.301 (*p* < 0.001)	[0.153, 0.436]
DIR Quality Grade vs. Suitability for Treatment Planning Grade	0.500 (*p* < 0.001)	[0.374, 0.608]

**Table 5:** Multiple linear regression model results predicting STP grades.

Criteria	Unstandardized Coefficient (B)	95% Confidence Interval for B	Standardized Coefficient (β)
Constant	0.996 (*p* < 0.001)	[0.541, 1.451]	-
Breathing Irregularity Grade	0.133 (*p* = 0.008)	[0.034, 0.231]	0.165 (*p* = 0.008)
FHFBCT Quality Grade	0.290 (*p* < 0.001)	[0.130, 0.449]	0.230 (*p* < 0.001)
DIR Quality Grade	0.404 (*p* < 0.001)	[0.292, 0.517]	0.467 (*p* <0.001)

**Table 6:** Multiple linear regression model results predicting mean model residual (mm).

Criteria	Unstandardized Coefficient (B)	95% Confidence Interval for B	Standardized Coefficient (β)
(Constant)	0.571 (*p* < 0.001)	[0.253, 0.888]	-
Breathing irregularity Grade	0.214 (*p* < 0.001)	[0.145, 0.282]	0.407 (*p* < 0.001)
FHFBCT Quality grade	0.147 (*p* = 0.010)	[0.035, 0.258]	0.177 (*p* = 0.010)
DIR Quality Grade	0.102 (*p* = 0.011)	[0.024, 0.181]	0.181 (*p* = 0.011)

**Table 7:** Multiple linear regression model results predicting 95th-percentile model residual (mm).

Criteria	Unstandardized Coefficient (B)	95% Confidence Interval for B	Standardized Coefficient (β)
(Constant)	0.758 (*p* = 0.012)	[0.170, 1.346]	-
Breathing irregularity Grade	0.279 (*p* < 0.001)	[0.152, 0.406]	0.281 (*p* < 0.001)
FHFBCT Quality grade	0.248 (*p* = 0.019)	[0.042, 0.455]	0.159 (*p* = 0.019)
DIR Quality Grade	0.412 (*p* < 0.001)	[0.266, 0.557]	0.384 (*p* < 0.001)

## Data Availability

Availability of the data used in this study is limited due to ongoing studies and restrictions on clinical data.
